# Comprehensive Metabolome Analysis of Fermented Aqueous Extracts of *Viscum album* L. by Liquid Chromatography−High Resolution Tandem Mass Spectrometry

**DOI:** 10.3390/molecules25174006

**Published:** 2020-09-02

**Authors:** Evelyn Peñaloza, Carla Holandino, Claudia Scherr, Paula I. P. de Araujo, Ricardo M. Borges, Konrad Urech, Stephan Baumgartner, Rafael Garrett

**Affiliations:** 1 Metabolomics Laboratory, Institute of chemistry, Federal University of Rio de Janeiro, 21941-598 Rio de Janeiro, Brazil; evelyn.cpenaloza@gmail.com (E.P.); ivenspaula@pos.iq.ufrj.br (P.I.P.d.A.); 2 Multidisciplinary Laboratory of Pharmaceutical Sciences, Faculty of Pharmacy, Federal University of Rio de Janeiro, 21941-902 Rio de Janeiro, Brazil; cholandino@pharma.ufrj.br; 3 Hiscia Research Institute, Society for Cancer Research, 4144 Arlesheim, Switzerland; c.scherr@vfk.ch (C.S.); k.urech@vfk.ch (K.U.); stephan.baumgartner@uni-wh.de (S.B.); 4 Walter Mors Institute of Research on Natural Products (IPPN), Federal University of Rio de Janeiro, 21941-902 Rio de Janeiro, Brazil; ricardo_mborges@yahoo.com.br; 5 Institute of Integrative Medicine, University of Witten/Herdecke, 58313 Herdecke, Germany

**Keywords:** mistletoe, metabolic profiling, LC-MS, host tree, metabolomics, natural products

## Abstract

Fermented aqueous extracts of *Viscum album* L. are widely used for cancer treatment in complementary medicine. The high molecular weight compounds viscotoxins and lectins are considered to be the main active substances in the extracts. However, a vast number of small molecules (≤1500 Da) is also expected to be present, and few studies have investigated their identities. In this study, a comprehensive metabolome analysis of samples of fermented aqueous extracts of *V. album* from two host tree species (*Malus domestica* and *Pinus sylvestris*), both prepared by two pharmaceutical manufacturing processes, was performed by liquid chromatography−high resolution tandem mass spectrometry (LC-HRMS/MS). A total of 212 metabolites were putatively annotated, including primary metabolites (e.g., amino acids, organic acids, etc.) and secondary metabolites (mostly phenolic compounds). A clear separation between *V. album* samples according to the host tree species, but not due to manufacturing processes, was observed by principal component analysis. The biomarkers responsible for this discrimination were assessed by partial least squares−discriminant analysis. Because *V. album* extracts from different host trees have different clinical applications, the present work highlights the possibility of characterizing the metabolome for identification and traceability of *V. album* fermented aqueous extracts.

## 1. Introduction

European mistletoe (*Viscum album* L., Santalaceae) is a perennial, hemi-parasite evergreen flowering plant [1,2,3]. It grows on the branches of different host trees (e.g., *Malus domestica*, *Pinus sylvestris*, *Quercus* spp., etc.) using a root-like structure called sinker, which penetrates into the xylem tissue of the host tree taking up water and mineral nutrients [2]. Different subspecies are reported, and they are morphologically distinguished according to their host trees: *V. album* L. ssp. *album* is found on deciduous trees, *V. album* L. ssp. *abietis* (Wiesb.) Abrom. occurs on fir, whereas *V. album* L. ssp. *austriacum* (Wiesb.) Volim. grows mainly on pine [3,4,5,6].

*V. album* has been used for centuries in European traditional medicine to treat many disorders, such as epilepsy, infertility, indigestion, hypertension, dizziness, and asthma [6]. However, only in the decade of 1920 it was introduced for cancer treatment by Steiner and Wegman [7]. After this, a boom in the amount of scientific reports was observed, for example botanical [2,3], phytochemical [5,8], pharmacological [6,8,9], pre-clinical [10], and clinical [11,12] studies. Many clinical studies demonstrated the efficacy of *V. album* extracts as adjuvant in cancer therapy to treat different types of tumors (gastrointestinal, breast, pancreas, lungs, kidneys, etc.), resulting in better survival rate, alleviation of conventional therapy side effects and improved quality of life, with very few adverse effects [12,13].

The first pharmaceutical preparation of *V. album* was developed based on a fermented aqueous extract. This preparation (Iscador^®^) is registered in several countries worldwide. Most of the clinical trials have been performed with this preparation, and there is broad evidence for its efficacy [12,14]. Preparations of *V. album* can be obtained from different host tree species and can be standardized by the quantity of lectin and/or dry/fresh extract of the whole plant [13,15].

The high molecular weight viscotoxins and mistletoe lectins are considered to be the main active substances present in the aqueous extracts of *V. album* [16]. However, it also contains small molecules, here defined as ≤1500 Da, such as phenolic acids, flavonoids, terpenoids, tannins, phytosterol, saponins, organic acids, etc. that may contribute to its biological activities [5,6,8,17]. In fact, there is some evidence that the pharmacological effects of *V. album* are due to the whole extracts rather than to lectins and viscotoxins only [18,19,20]. Despite this, the products commercially available are still poorly described concerning the small molecules composition.

The wide diversity of metabolites may vary qualitatively and quantitatively depending on the harvesting time, edaphoclimatic conditions, plant organs, growth stage, and extraction procedures. Moreover, as part of the chemical complexity of mistletoe, there is the influence of the host tree on the plant metabolome [5]. Its hemiparasite nature and dependence on nutritional resources from the host can alter the chemical profile of mistletoe. Thus, it becomes critical to comprehensively profile the chemical composition of *V. album* extracts in order to help improving their identification and traceability.

Liquid chromatography−mass spectrometry (LC-MS) is a powerful analytical technique used for the identification and quantification of a wide range of metabolites. Its advantages, such as sensibility, fast and feasible method for large scale sample analysis, make it the instrument of choice for plant-derived natural products untargeted metabolomics [21].

The objective of this work was to develop a liquid chromatography−high resolution tandem mass spectrometry (LC-HRMS/MS) approach to comprehensively profile and to identify biomarkers for fermented aqueous extracts of *V. album* grown on two different host trees (*M. domestica* and *P. sylvestris*) and processed by two manufacturing processes (anthroposophically processed *V. album* extract (APVAE) and common *V. album* extract (VAE)) [20].

## 2. Results and Discussion

### 2.1. LC-HRMS/MS Analysis and Compound Annotation

The LC-HRMS/MS method was developed combining full scan MS experiments with data-dependent fragmentation (DDA) in both positive (ESI+) and negative (ESI-) electrospray ionization modes to allow for the detection and putative identification of a wide range of metabolites. Samples (*n* = 68) were grouped into four groups, called ISCM (anthroposophically processed *V. album* extract (APVAE) from *M. domestica* host tree), ISCP (anthroposophically processed *V. album* extract (APVAE) from *P. sylvestris* host tree), HGM (common *V. album* extract (VAE) from *M. domestica* host tree), and HGP (common *V. album* extract (VAE) from *P. sylvestris* host tree).

A total of 212 metabolites were annotated in the fermented aqueous extracts of *V. album*, 28 by their high accurate *m*/*z* values (error ≤ 5 ppm for ESI+ and ≤ 10 ppm for ESI-, due to the *m*/*z* range of the calibrant solution used) and 184 by their high accurate *m*/*z* values and MS/MS spectra (MS level 2; metabolomics standards initiative, MSI) [22,23]. Their retention times (*t_R_*), adduct ions, experimental and theoretical *m*/*z* values, ppm error, MS/MS fragmentation spectra, SMILES (Simplified Molecular Input Line Entry System) string, and suggested compounds are available in supplementary Table S1. The annotated compounds described here were based on those available in public (e.g., https://mona.fiehnlab.ucdavis.edu/) or commercial databases (NIST 2014 MSMS library) as well as those already described in published articles for Viscum samples (Table S1).

From these metabolites, 120 were observed in the ESI+, 68 in the ESI-, and 24 were in common for both ionization modes. This highlights the importance of acquiring the MS data using both ionization modes to encompass a wide range of metabolites, from basic to neutral and acidic ones. Forty-two metabolites have already been described in Viscum samples (Table S1), including those 28 metabolites annotated only by MS (Table 1).

**Figure 1:**
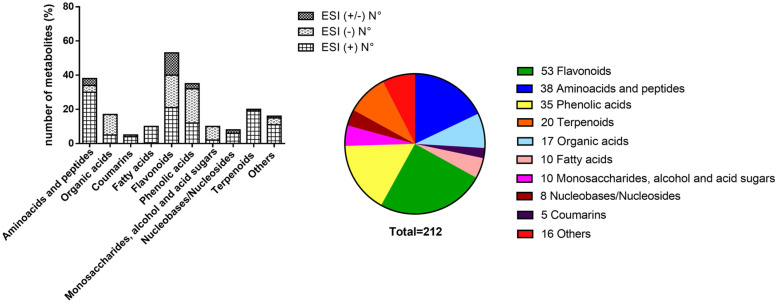
Metabolites in the fermented aqueous extracts of *V. album* putatively annotated by LC-HRMS/MS according to the ionization mode and chemical group.

A large number of high polar primary metabolites, such as amino acids, monosaccharides, sugar acids, sugar alcohols, peptides, organic acids, nucleobases, and nucleosides were found in the fermented aqueous extracts of *V. album*. Their distribution, according to the ionization modes and chemical classes, are shown in Figure 1. Because of the hemi-parasitic nature of this plant, it depends on water, minerals, and primary metabolites from the host via the xylem as a source of carbon and nitrogen for their development [2]. Thus, we cannot assure by the approach used whether these primary metabolites are produced by the *V. album* itself or may come from the host trees.

**Figure 2:**
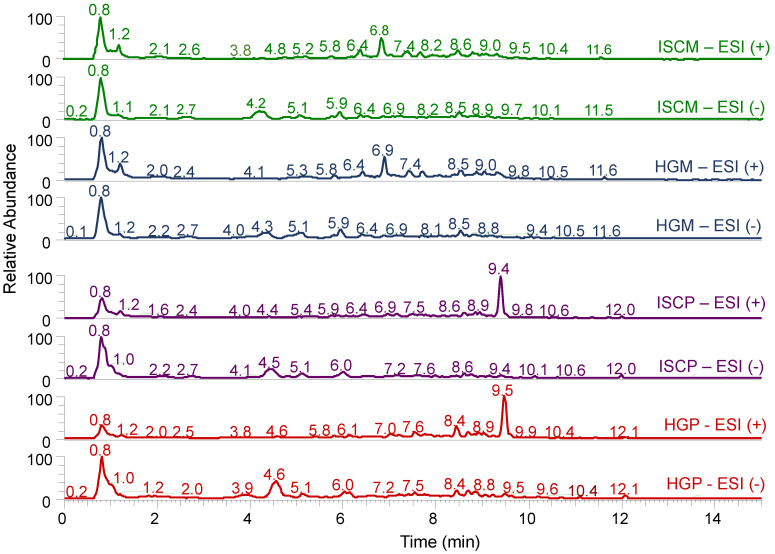
Typical LC-HRMS chromatograms of fermented aqueous extracts of *V. album* from the two host trees (*M. domestica* and *P. sylvestris*) and manufacturing processes (APVAE and VAE) in positive (ESI+) and negative (ESI-) electrospray ionization modes. Abbreviations: ISCM: *M. domestica*/APVAE, HGM: *M. domestica*/VAE, ISCP: *P. sylvestris*/APVAE; HGP: *P. sylvestris*/VAE.

Many of the annotated compounds eluted at the beginning of the chromatographic analysis, resulting in a large peak around 0.8 min. Typical LC-HRMS chromatograms of the fermented aqueous extracts of *V. album* from the two host trees (*M. domestica* and *P. sylvestris*) and manufacturing processes (APVAE and VAE) are shown in Figure 2. It is clear, by visual inspection, that the main differences among chromatograms from the same ionization mode were due to the difference in host trees rather than due to the manufacturing process.

Metabolites annotated in the extracts were classified as i) primary metabolites, such as amino acids and peptides (*n* = 38); organic acids (*n* = 17); monosaccharides sugar acids and sugar alcohols (*n* = 10); fatty acids (*n* = 10); nucleobases/nucleosides (*n* = 8); and ii) secondary metabolites, such as flavonoids (*n* = 53); phenolic acids (*n* = 35); terpenoids (*n* = 20); coumarins (*n* = 5); and other compounds (*n* = 16), as shown in the Figure 1.

Dipeptides were the most abundant compounds in the group of amino acids and peptides. A total of 23 dipeptides were annotated. They were observed in the ESI+ analysis as proton adducts and their MS/MS spectra showed losses of H_2_O and CO from the precursor ions. To the best of our knowledge, this class of compounds has never been described in Viscum samples. However, their presence may not be a surprise since these fermented aqueous extracts were rich in protein content. Examples of dipeptides found were Leu-Leu, Ile-Ile, Leu/Ile-Thr, etc. In addition, one tripeptide Leu/Ile-Pro-Leu/Ile with fragments at *m*/*z* 70.07, 86.10, 116.07, 183.15, and 211.14 was also annotated.

Organic acids were observed at the beginning of the chromatographic analysis. Their fragmentation was mainly characterized by the losses of water, carboxyl and/or hydroxyl groups. Monocarboxylic acids, such as ketogluconic acid and hydroxy-methylpentanoic acid; dicarboxylic acids, such as malic acid, meglutol, suberic acid, citramalic acid; and tricarboxylic acids, such as citric acid and aconitic acid were the main compounds. 1,5-dimethyl-6,8-dioxatricyclo [4.2.1.0]nonane-3-methyl-2,4-pentadienoic acid has already been described for *V. coloratum* [24].

Monosaccharides, sugar alcohols, and sugar acids were predominantly identified in the ESI-. Examples of annotated compounds were ribonolactone, levoglucosan (anhydro monosaccharide), sugar acids, such as threonic acid, galactonic acid, and phosphogluconic acid; and sugar alcohols, such as mannitol, inositol, pinitol, and viscumitol. The last three have already been identified in aqueous extracts of leaves of *V. album* [25,26]. It is noteworthy that Arda et al. [27] identified monosaccharides (glucose, galactose, mannose, arabinose, xylose) and poliols (xylitol and inositol) from two subspecies of *V. album* (ssp. *album* and ssp. *abietis*). They showed that glucose and galactose content were higher in *V. album* ssp. *abietis*, whereas *V. album* ssp. *album* possessed higher content of mannose, arabinose, and sugar alcohols. In the present work, *D*-ribose, inositol, and mannitol contents were higher in *V. album* samples hosted on *M. domestica,* as already described by Kohl et al. [28].

Previous studies have characterized nucleosides from aqueous extracts of *V. album* belonging to different hosts (e.g., *Pinus* sp. and *Malus* sp.) [29]. Some of them, 2′-desoxyadenosine, 2′-desoxyguanosine, and thymidine were found in our study. Furthermore, three nucleobases (guanine, adenine, and thymine) were annotated. They were eluted in the *t_R_* range of 0.80–1.17 min and ionized in the ESI+.

The predominant groups of phenolic compounds in the fermented aqueous extracts of *V. album* were hydroxybenzoic acids, hydroxycinnamic acids, lignans, and flavonoids and their derivatives (mainly glycosides and esters). Flavonoids (*n* = 53) were the most abundant class of such secondary metabolites, followed by hydroxycinnamic acids (*n* = 21), hydroxybenzoic acids (*n* = 10), and lignans (*n* = 4). Flavonoids, including many isomers and sugar derivatives, are the most common and widely distributed group of compounds in the plant kingdom. They possesses a vast range of pharmacological activities, have been described as anticarcinogenic agents, and protect the DNA against oxidative stresses [30]. This high number of flavonoids present in the extracts may indicate a contribution of this class of compounds to the Viscum pharmacological activity.

Prunin, eriodictyol, rhamnazin-3-*O*-*β-*d-(6″-*β*-hydroxy-*β*-methyglutaryl)glucoside, naringin, rhamnocitrin, and 5-hydroxy-3,7,3’-trimethoxyflavone-4’-*O*-*β*-d-glucoside, among others, have already been described in Viscum, such as *V. coloratum* [31], *V. schimperi* [32], *V. angulatum* [17], *V. articulatum* [33], and *V. liquidambaricola* [17]. The sugar moieties from flavonoid *O*-glycosides, such as hexoses, deoxy-hexose, pentoses, and uronic acid residues were identified by the neutral losses of 162 Da [C_6_H_10_O_5_], 146 Da [C_6_H_10_O_4_], 132 Da [C_5_H_8_O_4_], and 176 Da [C_6_H_8_O_6_], respectively.

The compounds 3-phenyllactic acid and dimethoxycinnamic acid showed high signal intensity in the *V. album* samples from *P. sylvestris* host tree. Twenty terpenoids and their derivatives were annotated, and three of them (carveol, loliolide, and vomifoliol) have already been described in *V. album* [34,35]. The anticancerogenic activities of terpenoids, such as loliolide, curcumol, dehydrovomifoliol and blumenol A have already been tested in tumor cell lines such as colon cancer, KB human oral epidermoid carcinoma cells, and HT29 colorectal carcinoma cells [36,37,38,39].

Other molecules were also detected in the fermented aqueous extracts of *V. album*, covering a wide variety of chemical classes. They also play critical roles in preventing and/or in the development of cancer. For instance, vitamin B is described as regulating the immune response [40]; benzoquinones acts against proliferation of cancer cells [41]; and alkaloids, such as swainsonine, possess therapeutic activity in patients with both solid tumor and hematological malignancies [42].

Considering the vast number and different classes of compounds present in the fermented aqueous extracts of *V. album*, one could expect that these small molecules may also be responsible or be adjuvant for the anticancer activities of Viscum plant extracts, and the chemical and biological studies of *V. album* may not be limited to the viscotoxins and lectins.

### 2.2. Multivariate Data Analysis

The processed and aligned LC-HRMS data were submitted to the principal component analysis (PCA) and partial least square−discriminant analysis (PLS-DA) multivariate analyses. The first one was used to assess natural sample variation, grouping and outlier detection, using the Hotelling’s T2 test with a confidence level of 95%. The second is a supervised technique and was employed for biomarkers identification of the natural groups identified in PCA. Two data matrices were used, one for data acquired in ESI- containing 75 samples × 949 variables (*m*/*z*_*t_R_*), and the other for ESI+ containing 75 samples × 1646 variables (*m*/*z*_*t_R_*). For PLS-DA, the pooled quality control (QC) samples were removed from the data matrix. Prior to multivariate analyses, data were normalized by the LOWESS (locally weighted scatterplot smoothing) method using the pooled QC samples and Pareto scaled to reduce the relative importance of variable with high intensities.

**Figure 3:**
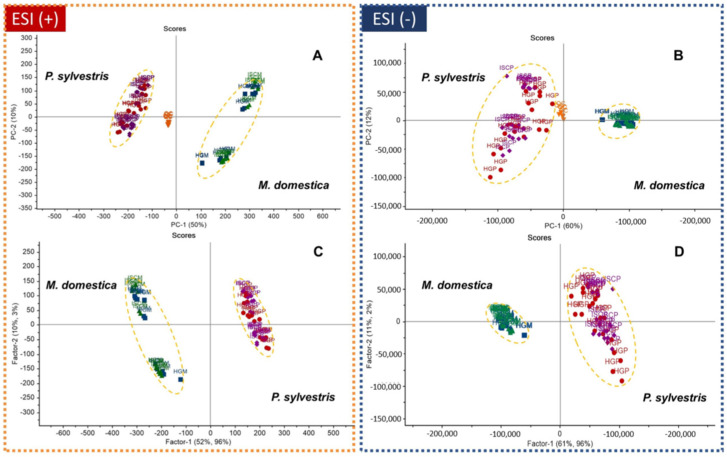
PCA (**A**,**B**) and PLS-DA (**C**,**D**) score plots from data of *V. album* fermented aqueous extracts analyzed by LC-ESI(±)-HRMS. Abbreviations: QC: pooled quality control samples, ISCM: *M. domestica*/APVAE, HGM: *M. domestica*/VAE, ISCP: *P. sylvestris*/APVAE; HGP: *P. sylvestris*/VAE.

PCA and PLS-DA score plots are shown in Figure 3. In PCA (Figure 3A,B), a clear separation between the *V. album* extracts according to the host tree (*M. domestica/P. sylvestris*) rather than the pharmaceutical processing method (VAE/APVAE) was observed. The pooled QC samples were tightly clustered, indicating the reproducibility of the LC-HRMS system. The same pattern was observed in the PLS-DA (Figure 3C,D). The first two principal components (or latent variables, for PLS-DA) in both analyses explained ≥60% of data variation.

The PLS-DA model was internally validated using full cross-validation. For the ESI+ data analysis, the coefficient of determination for calibration (R^2^cal) and cross-validation (R^2^val) were 0.995 and 0.991, respectively, whereas the root means square error of calibration (RMSEC) and cross validation (RMSECV) were 0.081 and 0.092, respectively. For the ESI- data analysis, the values were R^2^cal 0.991, R^2^val 0.984, RMSEC 0.0912, and RMSECV 0.123. These values indicated the reliability of PLS-DA models to predict sample classes.

**Figure 4:**
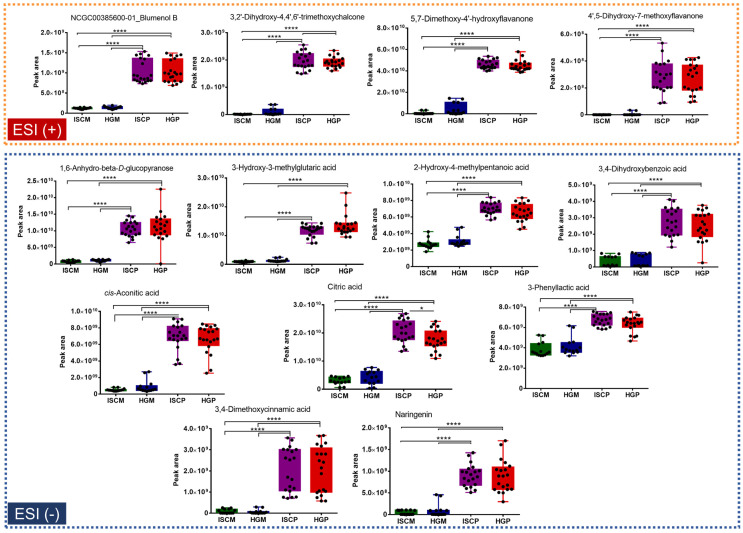
Box plot graphs of annotated metabolites responsible for the discrimination of *P. sylvestris* group from *M. domestica* in the PLS-DA model. Abbreviations: ISCM: *M. domestica*/APVAE, HGM: *M. domestica*/VAE, ISCP: *P. sylvestris*/APVAE; HGP: *P. sylvestris*/VAE. Tukey’s multiple comparison test was performed for significant difference. * *p* < 0.05; ** *p* < 0.01; *** *p* < 0.001; **** *p* < 0.0001.

**Figure 5:**
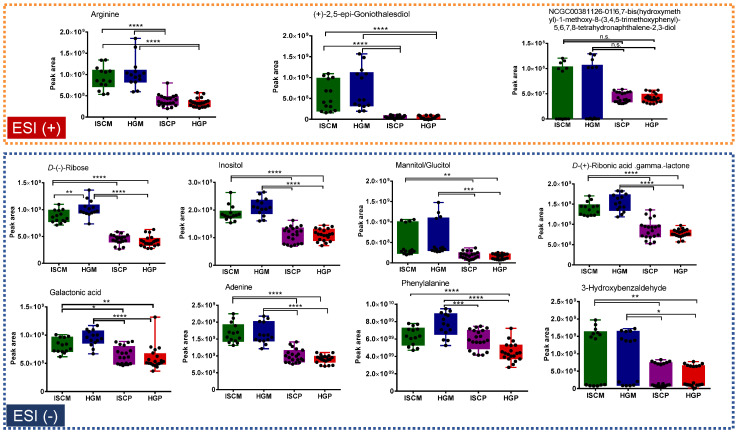
Box plot graphs of annotated metabolites responsible for the discrimination of *M. domestica* group from *P. sylvestris* in the PLS-DA model. Abbreviations: ISCM: *M. domestica*/APVAE, HGM: *M. domestica*/VAE, ISCP: *P. sylvestris*/APVAE; HGM: *P. sylvestris*/VAE. Tukey’s multiple comparison test was performed for significant difference. * *p* < 0.05; ** *p* < 0.01; *** *p* < 0.001; **** *p* < 0.0001. *n*.s. (not significant): *p* > 0.05.

Variables that were putatively annotated and mostly contributed to the sample class discrimination (*V. album* from *M. domestica* vs. *P. sylvestris* host trees), because of their high loading values in the PLS-DA analyses, are shown in Figure 4 and Figure 5. It is important to highlight that they are suggested compounds based on high-resolution and accurate MS and MS/MS spectra.

For the *P. sylvestris* group, variables (*m*/*z*_*t_R_* (min) ) that were present in higher amounts in the *V. album* from *P. sylvestris* compared to *M. domestica* in the ESI+ data analysis were blumenol B (209.15373_6.36), 3,2’-dihydroxy-4,4’,6’-trimethoxychalcone (331.1163_9.4), 5,7-dimethoxy-4’-hydroxyflavanone (301.10703_9.3), 4’,5-dihydroxy-7-methoxyflavanone (287.09024_7.65), whereas for ESI-, they were 1,6-anhydro- beta-D-glucopyranose (161.04431_0.8), 3-hydroxy-3-methylglutaric acid (161.04503_1.0), 2-hydroxy-4-methylpentanoic acid (131.06998_5.10), 3,4-dihydroxybenzoic acid (153.01814_1.7), *cis*-aconitic acid (173.00826_0.85), citric acid (191.0197_0.8), 3-phenyllactic acid (165.05479_5.9), 3,4-dimethoxycinnamic acid (207.0659_8.7), and naringenin (271.06177_9.1) (Figure 4).

For the *M. domestica* group, variables (*m*/*z*_*t_R_* (min) ) that were present in higher amounts in the *V. album* from *M. domestica* compared to *P. sylvestris* in the ESI+ data analysis were arginine (175.11873_0.7), (+) -2,5-*epi*-goniothalesdiol (249.11136_5.8), and NCGC00381126-01!6,7-*bis*(hydroxymethyl)-1-methoxy-8-(3,4,5-trimethoxyphenyl)-5,6,7,8-tetrahydronaphthalene-2,3-diol (421.18549_6.9), whereas for ESI- they were d- (−)-Ribose (149.04501_0.7), inositol (179.0549_0.7), mannitol/glucitol (181.07051_0.75), *d*- (+) -ribonic acid gamma.lactone (165.03972_0.7), galactonic acid (195.05043_0.78), adenine (134.04605_0.8), phenylalanine (164.0708_1.2), and 3-hydroxybenzaldehyde (121.02843_4.4) (Figure 5).

The results obtained support previous investigations carried out by Becker and Exner [43], Richter and Popp [25], Łuczkiewicz et al. [44], Pietrzak et al. [45], Jäger et al. [46], Gärtner et al. [47], and others, who demonstrated that the host trees play a major role shaping the metabolome of Viscum species. For example, Becker and Exner [43] investigated the methanolic extracts of *V. album* from different host trees by UV-spectroscopy, IR, ^1^H-NMR, and MS, and showed that flavonoids are differentially distributed among samples. Deliorman et al. [48] studying leaves, stems, and twigs from three *V. album* L. subspecies, showed that phenylpropanoids glycosides greatly varies among Viscum from different host trees. The variations of polyphenolics, such as rosmarinic acid and chlorogenic acid among Viscum samples, were also described by Łuczkiewicz et al. [44]. These two compounds were annotated in the present study. As noted, phenolic compounds are the small molecule compounds mostly described in Viscum samples.

### 2.3. Chemical Space by Molecular Networking

The LC-HRMS datasets from ESI+ and ESI- analyses used for multivariate analysis, together with a mascot generic format (MGF) file containing the MS/MS information of aligned spectra, were submitted to the feature-based molecular network workflow at the Global Natural Product Social Molecular Networking (GNPS) platform [49] for visualization of the chemical space of molecules and their distribution across sample groups [50]. Two molecular networks (MN) were created, one for each ionization polarity. They can be accessed at the GNPS web site via https://gnps.ucsd.edu/ProteoSAFe/status.jsp?task=b2c15f6337f54b2ba52b4546498e554d for the ESI-(+) network or https://gnps.ucsd.edu/ProteoSAFe/status.jsp?task=902e9ed5ba5444cfadf18570364da415 for the ESI-(–) network.

**Figure 6:**
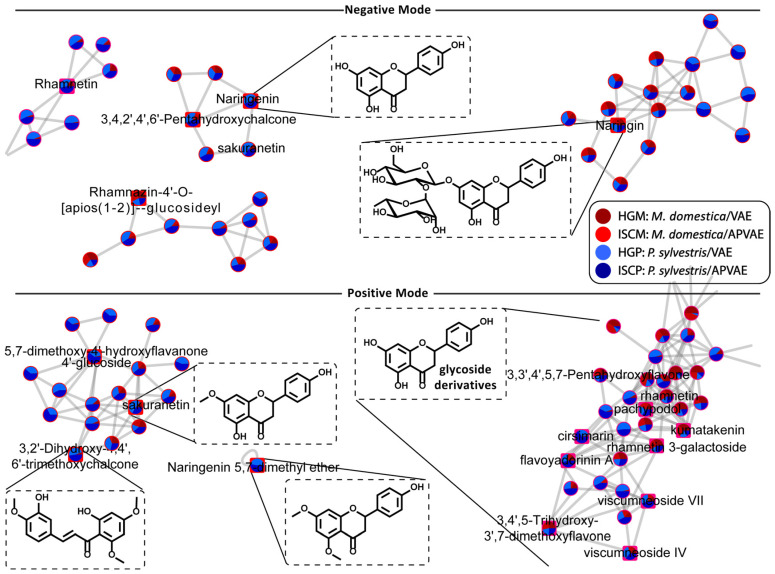
Nodes from the molecular network related to flavonoid structures obtained from fermented aqueous extracts of *V. album*.

The workflow MolNetEnhancer [51] was used to classify compounds within the networks. This workflow combines the output from the MN and other in silico workflows (not used in this study) to yield useful information for each MS/MS spectra to aid structure classification for each node. In practical terms, it enabled easy visualization of compound classes across the MN. By using this, it was possible to reinforce the results obtained by the manual compound annotation, where flavonoids were present in large number in the fermented aqueous extracts of *V. album* samples. The MN from the flavonoid class detected in both ESI(±) are shown in Figure 6. Since the clustering of compounds in the MN is based on the similarities of fragmentation spectra, we could assume that many unknown features present in the network shown in Figure 6 belonged to the flavonoid class. In general, the observed unknown features confirmed the complexity and vast number of small molecules present in these Viscum extracts. The clustering of annotated compounds in correct chemical classes according to their MS/MS spectra also reinforces that the suggestion of compound identity was consistent.

**Figure 7:**
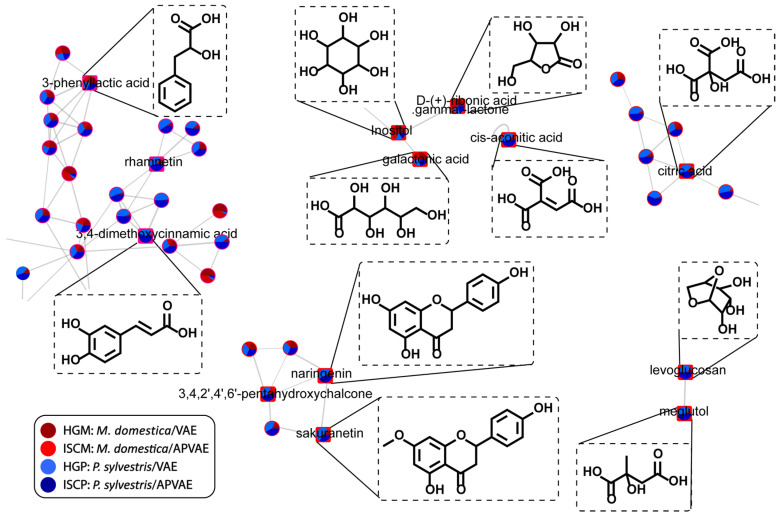
Nodes from the molecular network highlighting the compounds with high loading value in PLS-DA analysis and their distribution across different sample groups of fermented aqueous extracts of *V. album*.

The MN showing compounds described by PLS-DA as important for Viscum group discrimination is shown in Figure 7. It was possible to observe, for example, the clustering of free sugars with glycosylated flavonoids. The wide range of metabolites observed for *V. album* extracts represent an area that will require future efforts for a deeper annotation of the unknown metabolites.

## 3. Materials and Methods

### 3.1. Chemicals and Materials

Acetonitrile (HPLC/spectro grade) and formic acid (LC-MS grade) were from Tedia (Fairfield, USA). Water (18.2 MΩ∙cm) was from a Merck Millipore Milli-Q purification system.

Ampoules of 1.0 mL (200 mg·mL^−1^) of fermented aqueous extracts of *V. album* L. produced from whole mistletoe plants (one- to two-year-old leaves, stems and fruits) from two different host trees (*M. domestica* and *P. sylvestris*) and two manufacturing processes (APVAE and VAE) were obtained from Iscador AG (Arlesheim, Switzerland). The corresponding mistletoe plants originated from apple trees (*Malus domestica* Borkh.) growing on calcareous to argilliferous soils with an alkaline-to-neutral pH in the northwestern part of Switzerland as well as in France (Alsace), and from pine trees (*Pinus sylvestris* L.) growing on calcareous soil with an alkaline pH in the south of France (Alpes-de-Haute-Provence), respectively. No fertilizers were applied. Mistletoe from both host trees were harvested two times a year, before midsummer and at the end of the year. They were mechanically crushed and extracted by fermentation with mistletoe derived *Lactobacillus plantarum*. For APVAE, the fermented winter and summer mistletoe extracts were blended in a specific process on a high speed rotating disc. For VAE the two components were mixed without this specific blending process. More details about the preparation of APVAE and VAE were described elsewhere [20]. Thirty-four samples from different years of production and batches, were extracted and analyzed by LC-HRMS/MS. They were codified as: ISCM: *M. domestica*/APVAE (7 samples), HGM: *M. domestica*/VAE (7 samples), ISCP: *P. sylvestris*/APVAE (10 samples), HGP: *P. sylvestris*/VAE (10 samples).

### 3.2. Sample Preparation

The plant extracts were transferred from the ampoules to 1.5 mL centrifuge tubes, vortexed for 15 s, and centrifuged at 9000× *g* for 15 min (Thermo Scientific mySPIN 12 mini centrifuge). Then, 300 μL was mixed with 900 μL of acetonitrile, vortexed for 60 s, and incubated at −20 °C for 20 min for protein precipitation followed by centrifugation at 9000× *g* for 15 min. Then, 800 μL of the supernatant were removed, dried under nitrogen flow and stored at −20 °C until analysis. Before the LC-HRMS/MS analysis, samples were reconstituted using 250 µL of acetonitrile:water (15:85, *v*/*v*) and 10 μL for each sample was mixed to generate the pooled quality control (QC) sample. This QC sample was injected five times before randomized sample injection for column conditioning and at every 10 samples to evaluate the LC-HRMS/MS system performance.

### 3.3. LC-HRMS/MS Analysis

The chromatographic separation was performed on a Thermo Dionex UltiMate 3000 UHPLC system using a Thermo Hypersil Gold reversed phase C18 column (150 mm × 2.1 mm; 3.0 μm particle size). Mobile phase A was 0.1% formic acid and 5 mM ammonium formate in water, and mobile phase B was 0.1% formic acid in acetonitrile. The chromatographic separation was performed in gradient elution mode at a flow rate of 0.35 mL·min^−1^ as follows: 0–1 min B 5%, 1–16 min B 5–95%, 16–18 min B 95%, 18.1–22 min B 5%. The injection volume was 5 µL and the column temperature was 40 °C.

The LC system was coupled to a hybrid quadrupole-orbitrap QExactive Plus Mass Spectrometer (Thermo Scientific, Frenton, CA) equipped with an electrospray ionization source operating by switching between the positive and negative ionization modes. The source parameters were as follows: spray voltage: 3.9 kV (ESI+) and 3.3 kV (ESI-); capillary temperature: 320 °C; source temperature: 380 °C; S-lens RF level: 50; and sheath and auxiliary gases: 45 and 15 arbitrary units, respectively. Data were acquired in full scan over the range of *m*/*z* 120–1000 at a resolution of 35,000 FWHM (full width at half maximum) combined with data-dependent MSMS (DDA top 3) at a resolution of 17,500 FWHW, an isolation window of 1.2 Da and collision energy (NCE) of 15–45%.

### 3.4. Data Processing and Metabolite Identification

Raw data files were converted to ABF format using the Reifycs Abf (Analysis Base File) converter (https://www.reifycs.com/AbfConverter/). Then, ABF files were submitted to a metabolomics workflow using MS-DIAL software (RIKEN, version 3.98) [52] for data processing such as peak picking, deconvolution, peak alignment, and peak matching against a MS/MS library. Parameters used were as follows: MS1 and MS2 tolerances: 0.005 and 0.05 Da, respectively; minimum peak height: 500,000; mass slice width: 0.05; linear-weighted moving average as the smoothing method using 3 scans and peak width 5 scans; sigma window value for deconvolution: 0.4; and 0.1 min and 0.005 Da tolerances for peak alignment. Compound annotation was performed by comparing the aligned *m*/*z* ions (MS and MS/MS) to those available at the MassBank of North America (http://mona.fiehnlab.ucdavis.edu/), the NIST 2014 MS/MS library, the KNApSAcK platform [53] and the Dictionary of Natural Products [54]. Similarity score lower than 80% were discarded.

The aligned data tables from ESI(±) analyses and the MGF file containing the MS/MS data were exported from the MS-DIAL to the GNPS web server for feature-based molecular network (FBMN) analysis [55]. Parameters are described in Table S2. The workflow MolNetENhancer was also performed [51]. The networks were plotted using Cytoscape software (http://www.cytoscape.org) [56].

### 3.5. Statistical Analysis

The aligned data table was LOWESS normalized and exported from MS-DIAL software to Unscrambler^®^ X software (10.3, CAMO software AS, Norway) for multivariate statistical analysis. Data were Pareto scaled and submitted to the unsupervised method principal component analysis (PCA) and the supervised method partial least square-discriminant analysis (PLS-DA). The models were constructed using 5 PC and internally validated by full cross-validation. Additionally, hierarchical clustering heatmap and correlation analyses were performed in the online platform Metaboanalyst 4.0 (https://www.metaboanalyst.ca/), and their results are shown in the Supplementary Material (Figures S1–S4).

## 4. Conclusions

This study showed the potential of non-targeted metabolic profiling for the characterization of fermented aqueous extracts of *V. album* samples from different host trees and manufacturing processes. A total of 212 metabolites were annotated by MS and MS/MS analysis.

Lectins and viscotoxins are described as the most important compounds from mistletoe and are believed to be responsible for its pharmacological effects. However, small molecules may also play an important role in Viscum biological activity. Furthermore, they could act as chemical markers for species identification and provide an additional reference for quality control of *V. album* extracts. Thus, this work offers a valuable reference for further research into the mode of action of *V. album* extracts and development of manufacturing processes.

## Figures and Tables

**Table 1:** Number of metabolites putatively annotated in *Viscum album* samples by liquid chromatography−high resolution tandem mass spectrometry (LC-HRMS/MS).

Metabolites Annotation	ESI+	ESI-	ESI(±)	Total
-By MS and MS/MS, metabolites already reported in *V. album* species	13	20	9	42
-By MS and MS/MS, metabolites not previously reported in *V. album* species	93	42	7	142
-By MS only, metabolites already reported in *V. album* species	14	6	8	28
**Total**	120	68	24	212

## Supplementary Materials

The following are available online, Table S1. List of putatively identified compounds by LC-HRMS/MS in the fermented aqueous extract of *V. album* L. Table S2. GNPS parameters. Figures S1−S5.
